# Serum Levels of Carbamylated LDL and Soluble Lectin-Like Oxidized Low-Density Lipoprotein Receptor-1 Are Associated with Coronary Artery Disease in Patients with Metabolic Syndrome

**DOI:** 10.3390/medicina55080493

**Published:** 2019-08-15

**Authors:** Teodora Stankova, Ginka Delcheva, Ana Maneva, Stefka Vladeva

**Affiliations:** 1 Department of Biochemistry, Faculty of Pharmacy, Medical University—Plovdiv, 4002 Plovdiv, Bulgaria; 2 Clinic of Endocrinology and Metabolic Disorders, University Hospital “Kaspela”, 4001 Plovdiv, Bulgaria

**Keywords:** carbamylated LDL, soluble lectin-like oxidized LDL receptor-1 (LOX-1), metabolic syndrome, coronary artery disease, serum biomarkers, lipoprotein modifications

## Abstract

*Background and objectives*: Lectin-like oxidized low density lipoprotein receptor-1 (LOX-1) has been recognized as the primary receptor for carbamylated low-density lipoproteins (cLDL) and is increasingly being viewed as a critical mediator of vascular inflammation and atherosclerosis. The aim of the current study was to evaluate the possible role of circulating cLDL and soluble LOX-1 (sLOX-1) as potential biomarkers of metabolic syndrome (MetS) as well as of coronary artery disease (CAD) among MetS patients. *Materials and Methods*: The serum levels of cLDL and sLOX-1 were measured by ELISA in 30 MetS patients without CAD, 30 MetS patients with CAD, and 30 healthy controls. *Results*: Patients with MetS had significantly higher serum levels of both cLDL and sLOX-1 than the healthy controls but lower in comparison to MetS + CAD subjects. Serum sLOX-1 concentration correlated significantly with fasting glucose (r**_s_** = 0.414, *p* = 0.001) and high-density lipoprotein (HDL)-cholesterol (r**_s_** = −0.273, *p* = 0.035) in the whole MetS cohort, whereas it correlated with cLDL only in the MetS + CAD subgroup (r**_s_** = 0.396, *p* = 0.030). The receiver-operating characteristic (ROC) curves of cLDL and sLOX-1 for MetS diagnosis had area under the curve (AUC) values of 0.761 and 0.692, respectively. AUC values of cLDL and sLOX-1 for CAD diagnosis among MetS patients were 0.811 and 0.739. Elevated serum levels of cLDL and sLOX-1 were associated with a higher risk of MetS development [odds ratio (OR) 24.28, 95% confidence interval (CI): 5.86–104.61, *p* < 0.001 and OR 4.75; 95% CI: 1.58–14.25, *p* = 0.009] as well as with presence of CAD among MetS subjects (OR 11.23; 95% CI: 3.10–40.71, *p* < 0.001 and OR 4.03; 95% CI: 1.73–11.84, *p* = 0.019, respectively). *Conclusions*: The present study underscores the potential of cLDL and sLOX-1 as promising biomarkers for diagnosis and risk assessment of MetS and CAD among the MetS population.

## 1. Introduction

Metabolic syndrome (MеtS) represents a cluster of metabolic abnormalities, including visceral obesity, hypertension, hyperglycemia and atherogenic dyslipidemia—hypertriglyceridemia, and low levels of high-density lipoprotein (HDL) cholesterol [1]. Although the additive predictive value of MetS components for cardiovascular diseases (CVD) is still debated, large prospective investigations have concluded that MetS increases the relative risk for coronary artery disease (CAD) by about two times, even in the absence of baseline CVD and diabetes mellitus (DM) [2,3,4]. Both morbidity and mortality from CAD and other cardiovascular causes are higher in patients with MetS. Hence, early assessment of CAD risk among people with MetS is desirable because it could ameliorate clinical outcomes [5]. However, the conventional cardiovascular risk factors such as smoking, hypertension, diabetes, and hypercholesterolemia explain only part of the risk of developing atherosclerosis. A growing body of evidence has underscored the key role of the interplay between modified low-density lipoproteins and their scavenger receptors in the pathogenesis and the progression of atherosclerosis and atherosclerosis-related diseases [6]. Several proatherogenic modifications of low-density lipoprotein (LDL) have been detected in humans, and oxidized LDL (oxLDL) is the best characterized [6]. Increased circulating levels of oxLDL have been associated with higher incidence of MetS [7] and CAD in the general [8] and in the MetS population [9]. Despite the evidence that LDL is also subjected to carbamylation in vivo [10], there have been no investigations of carbamylated LDL (cLDL) in patients with MetS.

Carbamylation of LDL is a result of the spontaneous nonenzymatic binding of isocyanate to the N-terminal protein amine groups or to multiple lysine residues of apolipoprotein B. The latter case yields ε-carbamyllysine, also called homocitrulline (HCit) [10,11]. Isocianate is the active form of urea-derived cyanate, which is normally present in low concentrations in plasma and is significantly increased in renal failure [11]. Accordingly, the pathological impact of carbamylation has been predominantly investigated in patients with chronic kidney disease (CKD). Intriguingly, recent studies have shown that chronic inflammation and oxidative stress, both highly implicated in the pathogenesis of MetS and atherosclerosis, are also mechanistically linked to promotion of protein carbamylation. Wang et al. revealed an alternative urea-independent pathway for carbamylation mediated by myeloperoxidase (MPO) [12]. MPO is a heme containing enzyme abundant in the granules of neutrophils, monocytes, and certain tissue macrophages such as those found in human atheroma [13]. At inflammatory sites and in atherosclerotic plaques, MPO catalyzes the oxidation of thiocyanate in the presence of hydrogen peroxide, thus producing isocyanate. The role of MPO in carbamylation in vivo has been further confirmed by studies with MPO knock-out and MPO transgenic mice [12].

Therefore, carbamylation is increasingly being considered as an additional contributory factor in the pathophysiology of CVD. Moreover, Apostolov et al. reported that cLDL is the quantitatively dominant LDL isoform not only in uremic patients but also in healthy individuals, proposing its possible role in atherosclerosis [14]. Indeed, in vitro studies have shown that cLDL harvests all the major proatherogenic activities, such as induction of endothelial dysfunction and endothelial cell apoptosis, macrophage foam-cell formation, increased adhesion molecule expression, and vascular smooth muscle cell proliferation [10,15,16].

The lectin-like oxidized low-density lipoprotein receptor-1 (LOX-1) has recently been recognized as the primary receptor for cLDL. LOX-1 is a class E scavenger receptor first identified as the major mediator of oxLDL biological effects in endothelial cells [17]. Subsequent studies have demonstrated that LOX-1 is also expressed by other cells highly related to atherosclerosis development, such as monocytes/macrophages, vascular smooth muscle cells (SMCs), cardiomyocytes, and platelets. Thus, LOX-1 has been suggested as a critical player in vascular inflammation and in atherosclerotic plaque formation, destabilization, erosion, and rupture [18]. LOX-1 expressed on the cell surface can be proteolytically cleaved at its membrane proximal extracellular domain and released as a soluble form (sLOX-1) [19]. It has been demonstrated that sLOX-1 may reflect the expression of membrane-bound LOX-1, and serum sLOX-1 levels are elevated in patients with acute coronary syndrome (ACS) [20]. In addition, sLOX-1 has been suggested as a new biomarker in the diagnosis and the prognosis of CAD [21]. Increased sLOX-1 concentrations have also been detected in patients with obesity [22] and type 2 DM [23], other medical conditions closely related to MetS.

The expression of LOX-1 is not constitutive but can be dynamically controlled. Glucose, oxLDL, shear stress, proinflammatory cytokines, and angiotensin II have been shown to induce LOX-1 expression in cultured endothelial cells and macrophages as well as in vivo [18,23,24,25]. In contrast, only in vitro studies have demonstrated that LOX-1 receptor can be upregulated by cLDL [17].

The provided data suggest involvement of carbamylation and LOX-1 in the pathogenesis of atherosclerosis. Nevertheless, to the best of our knowledge, there is no study revealing the potential role of serum cLDL and sLOX-1 in MetS with and without cardiovascular complications.

The aim of the current study was to investigate the association between MetS, cLDL, and LOX-1. The possible role of circulating cLDL and sLOX-1 as potential biomarkers for CAD in MetS patients was also evaluated. Therefore, the serum concentrations of cLDL and sLOX-1, the relationship between them, and the correlations with MetS components and some other classical cardio-metabolic risk factors were analyzed.

## 2. Materials and Methods

***Subjects***: This preliminary cross-sectional study involved 30 patients with MеtS and 30 patients with MеtS complicated with CAD. All MetS subjects were admitted to the Clinic of Endocrinology and Metabolic Disorders, University Hospital “Kaspela”, Plovdiv, Bulgaria. Patients were diagnosed as having MetS according to the International Diabetes Federation (IDF) global consensus definition modified by the joint statement of the National Heart, Lung and Blood Institute, the American Heart Association, the World Heart Federation, the International Atherosclerosis Society, and the International Association for the Study of Obesity. The presence of any three or more of the following five parameters was considered as diagnostic of MetS: (1) waist circumference of ≥94 cm in men, ≥80 cm in women; (2) elevated triglyceride (TG) levels ≥1.7 mmol/L or drug treatment for elevated TG; (3) reduced HDL-cholesterol of <1.0 mmol/L in men, <1.3 mmol/L in women or history of specific treatment for this lipid abnormality; (4) elevated blood pressure (BP), systolic BP ≥130 mmHg or diastolic BP ≥85 mmHg or drug treatment for hypertension; and (5) fasting blood glucose (FG) >5.6 mmol/L or drug treatment for hyperglycemia [1].

The enrollment of MetS patients in the MetS + CAD group was based on a documented medical history of CAD (myocardial infarction, classic angina pectoris, coronary artery bypass graft surgery, percutaneous coronary intervention, or a stenosis of 50% or greater in one or more major coronary vessels on angiography). The presence of CAD was confirmed by an electrocardiogram and/or angiogram.

The control group consisted of 30 healthy, sex-matched individuals who had no history of impaired glucose tolerance, DM, CAD, or other CVD. The control subjects were not taking any medications or antioxidant supplements. All the enrolled participants met the following exclusion criteria: any acute cardiovascular events during the last 3 months before hospitalization, renal or liver diseases, acute or chronic infections or inflammatory diseases, immunologic diseases or malignancies, other known chronic illness, pregnancy, or alcohol abuse. Individuals on statin therapy and current smokers were also not recruited in the study.

The study was approved by the Human Ethics Committee of Medical University – Plovdiv (N°4/21.09.2017) and was conducted in accordance with the Declaration of Helsinki, as revised in 2013. All participants signed an informed consent.

***Laboratory analysis***: Clinical data and fasting blood samples were collected. Serum FG, total cholesterol (TC), and TG were determined enzymatically on a Beckman Coulter AU480 analyzer (Beckman Instruments Inc., Brea, CA, USA). HDL-cholesterol was measured using a direct method with polyethylene glycol-modified enzymes and alpha-cyclodextrin. LDL-cholesterol was calculated using the Friedewald’s equation: [LDL-cholesterol] = [TC] − [HDL-cholesterol] − ([TG]/2.2) where all concentrations are given in mmol/L. Serum urea was measured by kinetic urease/glutamate dehydrogenase method and creatinine by the Jaffe method. Serum concentrations of cLDL and sLOX-1 were determined using commercially available ELISA kits (MyBioSource Inc., San Diego, CA, USA). Commercial ELISA kits were also used for quantification of serum levels of high-sensitivity C-reactive protein (hs-CRP) (BioVendor—Laboratorni medicina, Brno, Czech Republic). All the tests were performed according to the manufacturer’s instructions. The intra- and the inter-assay coefficients of variance were less than 9%.

***Statistics***: Statistical analysis was performed using SPSS software, version 17.0 (SPSS Inc., Chicago, IL, USA). The Kolmogorov–Smirnov test was used to evaluate whether the distribution of continuous variables was normal. Continuous variables were expressed as mean ± SD or as median and interquartile range. Analysis of normally distributed variables included an independent-samples t-test and one-way analysis of variance (ANOVA). The Mann–Whitney U-test and the Kruskal–Wallis test were used for variables with a non-normal distribution. As more conservative methods, t- or Mann–Whitney U-tests with Bonferroni corrections for multiple comparisons were preferred to the post-hoc tests in the assessment of the differences among the three studied groups. Categorical variables were analyzed by a Chi-square test. To address the relationships between variables, Spearman’s correlations were performed. Sensitivity and specificity of cLDL and sLOX-1 were analyzed using receiver operating characteristic (ROC) curves. A logistic regression analysis was conducted to evaluate the relationships between the studied biomarkers and the outcomes. The results are expressed as odds ratios (OR) with 95% confidence intervals (95% CI). The level of significance was set at *p* < 0.05.

## 3. Results

The baseline clinical characteristics of the study participants are summarized in Table 1.

**Figure 1:**
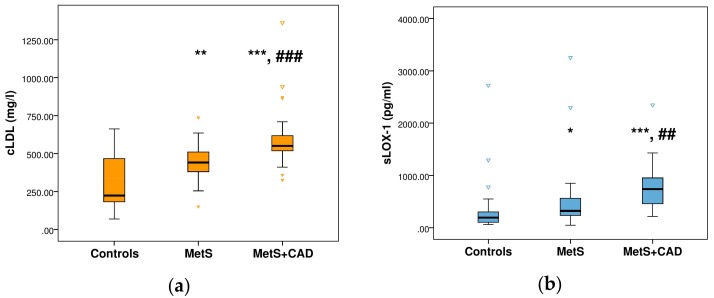
Serum concentrations of (**a**) cLDL and (**b**) soluble lectin-like oxidized LDL receptor-1 (sLOX-1) in control and MetS groups with and without CAD. Significance level versus control at * *p* < 0.05, ** *p* < 0.01 and *** *p* < 0.001, respectively. Significance level versus MetS group at: **##** *p* < 0.01 and **###** *p* < 0.001, respectively.

There were no significant differences among the three groups in regards to gender and serum levels of urea, creatinine, HDL-cholesterol, and TG, though the latter tended to be higher in the two patient groups when compared to the healthy volunteers. MetS + CAD patients were older only than the controls (*p* = 0.012). Both MetS groups with and without CAD had higher body mass index (BMI), waist circumference (WC), total cholesterol, and systolic and diastolic blood pressure (BP) than the controls. However, only MetS subjects without CAD showed higher concentrations of LDL-cholesterol (*p* < 0.001), while the MetS + CAD group had higher concentrations of FG in comparison to healthy subjects (*p* = 0.010). As expected in a MetS population, the circulating levels of hs-CRP were elevated in both MetS subgroups in comparison to controls. In contrast, the presence of CAD resulted only in a tendency for higher serum levels of hs-CRP in the MetS + CAD group than in their MetS counterparts (*p* = 0.035, but due to Bonfferoni correction for multiple comparisons, *p* < 0.016 was considered statistically significant). In addition, the two MetS groups were similar in terms of all other baseline parameters (Table 1). However, significant differences were observed in the serum concentrations of cLDL and sLOX-1 among the three studied groups. The highest serum levels of both cLDL (Figure 1a) and sLOX-1 (Figure 1b) were registered in MetS + CAD, followed by MetS and control groups.

Correlation analysis was further performed in the whole patient and in control groups. Serum sLOX-1 levels were found to correlate significantly and positively with FG, while they correlated negatively with HDL-cholesterol within the whole MetS cohort (Table 2). Serum sLOX-1 levels also tended to correlate positively with BMI, systolic BP, and hs-CRP in all MetS patients (Table 2).

**Figure 2:**
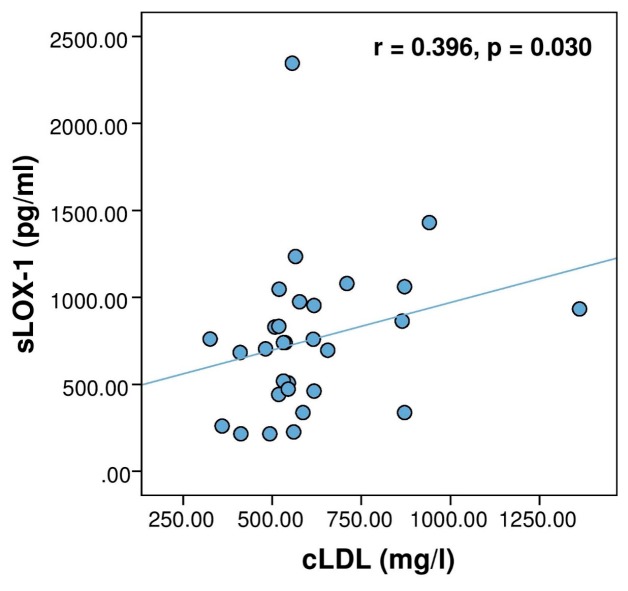
Correlation between the serum concentrations of sLOX-1 and carbamylated low-density lipoprotein (cLDL) in patients with metabolic syndrome and CAD (*n* = 30).

In addition, a significant moderate correlation between the serum levels of cLDL and sLOX-1 was established within the whole MetS cohort (r**_s_** = 0.420, *p* = 0.001, *n* = 60) but not in the healthy subjects (r**_s_** = −0.146, *p* = 0.441). We wanted to explore further whether the presence of CAD affected the magnitude of this novel correlation. Interestingly, when cLDL/sLOX-1 correlation was analyzed in each MetS subgroup, it remained significant only within MetS + CAD patients (Figure 2) but not in the MetS subjects without CAD (r**_s_** = 0.288, *p* = 0.123).

Another interesting finding is that cLDL correlated positively with urea only in controls (r**_s_** = 0.550, *p* = 0.002). No other significant correlations of cLDL and sLOX-1 were detected in both case and control groups.

**Figure 3:**
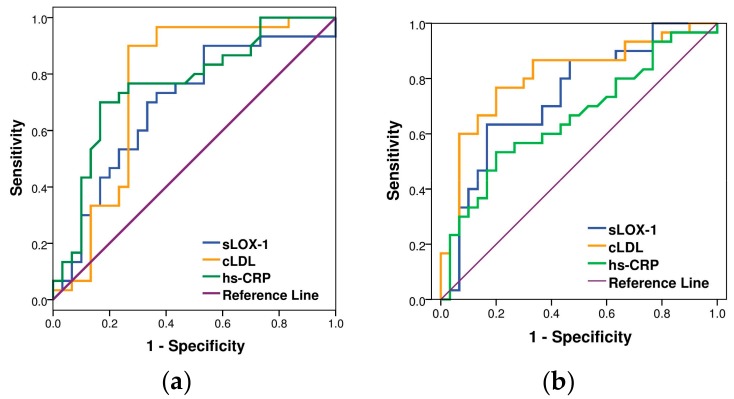
Receiver operating characteristics (ROC) curves of sLOX-1, cLDL, and hs-CRP for diagnosis of: (**a**) MetS among control and MetS subjects without CAD and (**b**) CAD among patients with metabolic syndrome. True-positive fraction (sensitivity as y axis) was plotted vs. false positive fraction (1-specificity as x axis) by changing cutoff values for test.

Based on the significant differences of cLDL and LOX-1 levels among the three studied groups, we hypothesized their potential role as diagnostic tools for MetS as well as for CAD among the MetS population. To test this hypothesis further, ROC analysis was conducted among the healthy controls and uncomplicated with CAD MetS subjects (Figure 3a) as well as among MetS patients with and without CAD (Figure 3b). We compared the diagnostic sensitivity and the specificity of sLOX-1 and cLDL to these of hs-CRP as one of the most commonly used biomarkers for MetS and CAD risk assessment [3].

The data about the diagnostic sensitivity and the specificity of sLOX-1, cLDL, and hs-CRP, the area under the curve (AUC), and the optimal cutoff values for the diagnosis of MetS and of CAD among MetS are summarized in Table 3 and Table 4, respectively. Our results indicate that sLOX-1 had comparable—and cLDL had even more remarkable—sensitivity and specificity in regards to MetS diagnosis when compared to hs-CRP (Figure 3a and Table 3). In addition, serum levels of sLOX-1 and cLDL could discriminate CAD among MetS patients even better than hs-CRP (Figure 3b and Table 4). In both cases, cLDL showed superior diagnostic potential compared to sLOX-1.

These observations were further confirmed by a logistic regression analysis. Serum sLOX-1 and cLDL levels above the aforementioned cutoff values (Table 3 and Table 4) were significantly associated with a higher risk of MetS (OR 4.75; 95% CI: 1.58–14.25, *p* = 0.009 and OR 24.28, 95% CI: 5.86–104.61, *p* < 0.001, respectively). Moreover, increased sLOX-1 and cLDL were significantly associated with the presence of CAD among MetS subjects (OR 4.03; 95% CI: 1.73–11.84, *p* = 0.019 and OR 11.23; 95% CI: 3.10–40.71, *p* < 0.001, respectively).

## 4. Discussion

A potential role for carbamylation in human health and disease has thus far been investigated solely in the context of CKD, as uremia provides a favorable chemical environment for this posttranslational modification (PTM) [11]. Protein carbamylation, and cLDL in particular, has even been suggested as a nontraditional risk factor in patients with CKD, explaining the higher prevalence and the severity of CVD in this subpopulation [10,15,26]. Recently, the discovery of a novel urea-independent and MPO-mediated mechanism for carbamylation has provided a vital insight into this PTM, linking it to smoking, inflammation, and atherosclerosis [12]. Although it is well-established that MetS is a prominent risk factor for CAD, and inflammation is involved in the pathogenesis of both of MetS and atherosclerosis, the role of carbamylation in MetS has not been elucidated yet.

The main finding of the current study was that patients with MetS without renal impairment had higher serum levels of cLDL than the healthy controls, but lower in comparison to MetS + CAD group. This observation may at least partially explain the higher cardiovascular burden in the MetS subpopulation, since cLDL has been shown to possess all the major proatherogenic activities, including binding to macrophage scavenger receptors, promoting cholesterol accumulation, and foam cell formation [17]. Carbamylated LDL causes endothelial cell apoptosis [15] as well as accelerated senescence in human endothelial progenitor cells [27]. Furthermore, cLDL potentiates atherosclerotic plaque formation and progression by stimulating proliferation of vascular SMCs and also by increasing the expression of cell adhesion molecules [16].

Our data even underscored serum cLDL levels as a reliable marker with superior diagnostic sensitivity and specificity compared to the commonly used hs-CRP in the identification of MetS among healthy controls and CAD among MetS patients [3,28]. Thus, our results are in accordance with two separate clinical studies that delineated plasma levels of protein-bound Hcit, another marker of carbamylation, as an independent predictor of an increased risk of CAD, future myocardial infarction, stroke, and death in 1000 participants from the general population. Of note, Hcit concentration predicted CVD incidents even after extensive adjustments for traditional cardiovascular risk factors, renal function, and both MPO and hs-CRP levels [12].

These adverse effects of cLDL are mainly attributed to their interaction with LOX-1, which is increasingly being viewed as a mediator of endothelial dysfunction, vascular inflammation, and atherosclerotic plaque formation [29]. LOX-1 has been shown to be proteolytically cleaved at the membrane proximal extracellular domain and released from the cell surface as sLOX-1 [19]. The level of circulating soluble receptors may reflect the expression of membrane proteins; hence, sLOX-1 might be a potential biomarker of vascular diseases. Recent lines of evidence reporting that LOX-1 is upregulated by MetS components have suggested a potential role of sLOX-1 also as a biomarker of metabolic disorders [30]. Indeed, our patients with MetS had higher sLOX-1 levels than the healthy controls. Despite being statistically significant, our ROC analysis of serum sLOX-1 as a diagnostic tool for MetS showed both lower sensitivity and specificity than reported in another study [30]. In addition, Puttaruk et al. documented that sLOX-1 correlated significantly with all MetS components [30], whereas we registered only a positive correlation with glucose, a negative correlation with HDL-cholesterol, and a tendency for positive correlations with systolic BP and BMI. These results are in line with several in vitro and animal investigations showing that, while the baseline LOX-1 expression is low in healthy endothelium, hypertension [31], hyperglycemia [32], and dyslipidemia [33] have been reported to increase it. Moreover, circulating levels of sLOX-1 have also been found to increase in obesity [22], type 2 DM [23], and even to correlate positively with reduction in body weight [34].

Although sLOX-1 initially emerged as a novel marker for ACS with a prognostic capacity exceeding that of hs-CRP and troponin-T [20], Lubrano et al. proposed sLOX-1 also as a marker of CAD [21]. Our results for higher sLOX-1 levels in the MetS group with CAD in comparison to MetS without CAD are in line with other studies reporting elevated sLOX-1 concentrations in CAD [35]. Nonetheless, the majority of the studies have explored the impact of LOX-1 on the incidence of CAD. In contrast to them, we wanted to elucidate the role of LOX-1 in the development of MetS, but, on the other hand, we also tried to evaluate the risk of CAD development on the background of existing MetS. Our observation that the concentration of sLOX-1 increased gradually from controls through MetS patients, being the highest in the MetS + CAD subgroup, is in concordance with a clinical study conducted by Sayed et al. [36]. Furthermore, our subsequent ROC analysis outlined that sLOX-1 was also able to detect CAD in MetS subjects. Serum sLOX-1 concentrations above the cutoff value of 495 pg/mL were associated with a four-fold higher relative risk of developing CAD among the MetS population. In addition, a recent investigation related higher sLOX-1 levels to the severity of CAD in patients with MetS [37]. Increased serum sLOX-1 have also been associated with arterial stiffness [38], coronary in-stent restenosis in patients with stable CAD [39], as well as with an increased risk of periprocedural myocardial infarction in stable CAD patients undergoing elective percutaneous coronary intervention [40]. Several lines of evidence have revealed that elevated sLOX-1 is not only restricted to acute coronary events but is also observed in acute ischemic stroke [41]. Taken together, our results indicate that patients with MetS are at a higher risk for future cardiovascular incidents, and this risk is the highest in subjects with MetS complicated with CAD.

The interaction between the increased levels of cLDL and LOX-1 might contribute to the augmented cardiovascular risk. Carbamylated LDL has been shown to upregulate LOX-1 expression in endothelial cells [17], but there is no evidence confirming this effect in vivo. Many recent investigations have demonstrated correlations between serum concentration of soluble LOX-1 and its other ligands in different pathological conditions [23,35]. Therefore, we hypothesized that circulating sLOX-1 may also be associated with cLDL in MetS. However, a significant weak correlation was observed only in the MetS + CAD subgroup. Other modified lipoprotein complexes have also been shown to upregulate LOX-1 expression, reflected by the soluble form of the receptor [35,42].

It is well-established that elevated oxLDL in plasma and within atherosclerotic lesions is strongly associated with CAD, ACS, and vulnerable plaques [8]. The atherogenicity of oxLDL is mainly due to its interaction with LOX-1, the major endothelial scavenger receptor for both oxLDL and cLDL, expressed also in SMCs and macrophages, the other major cell types involved in atherogenesis [18]. Hence, it has been proposed that cLDL-LOX-1 interaction elicits similar adverse effects on cardiovascular homeostasis. Indeed, Speer et al. demonstrated that binding of cLDL to LOX-1 in endothelial cells leads to p38 mitogen-activated protein kinase (MAPK) and NADPH oxidase activation. Carbamylated LDL stimulates endothelial nitric oxide (NO) synthase (eNOS) uncoupling via S-glutathionylation and also inhibits eNOS activation by directly affecting its phosphorylation [43]. These downstream effects of cLDL-LOX-1 interaction lead to reactive oxygen species (ROS) overproduction, decreased NO bioavailability, and induction of endothelial dysfunction [43,44]. The increased generation of ROS on their turn can upregulate LOX-1 expression, establishing a vicious cycle between LOX-1 and ROS [45]. Oxidative stress and endothelial dysfunction have been accepted as the common key players in the pathogenesis of both MetS and its cardiovascular complications. Therefore, our data indicate that cLDL-LOX-1 interplay might also be involved in the crosstalk between MetS and CAD.

Another interesting finding of our study is that cLDL correlated significantly with urea only in the healthy controls, but no such correlation was observed in both MetS subgroups. Considering the fact that only urea- and MPO-mediated mechanisms of carbamylation have been documented [11,12], we might speculate that cLDL is generated mainly via the nonenzymatic urea-dependent pathway under normal physiological conditions, whereas via the alternative MPO-mediated mechanism in the pathophysiological conditions of MetS complicated or uncomplicated with CAD. Furthermore, augmented MPO levels have been associated with obesity, chronic low-grade inflammation, insulin resistance, MetS, and type 2 DM [13,46]. In addition, increased MPO levels have been shown to predict the future risk of CAD in apparently healthy individuals, indicating that MPO-catalyzed inflammatory activation precedes the onset of overt CAD by many years [47]. Intriguingly, we registered the highest cLDL concentration in the MetS + CAD cohort. The hypothesis for MPO-driven LDL carbamylation in type 2 DM was previously confirmed by Shiu et al. [48] as well as by our research team. We established a positive significant correlation between cLDL and 3-nitrotyrosine, considered as a molecular fingerprint of MPO-catalyzed oxidation, in poorly controlled type 2 DM [49]. However, MPO measurement was not a subject of the current study, and the mechanism of LDL carbamylation in MetS merits further investigation.

### Limitations

Our study is subject to certain limitations. First, the small number of the included subjects in each group is the biggest limitation of the present study. Second, because of its cross-sectional design, we could only demonstrate mechanistic associations but not causal relationships. Third, although we avoided the confounding effect of smoking on cLDL levels, diet is another source of thiocyanites that can fuel the MPO-mediated carbamylation pathway. Moreover, besides the conventional medicines, some natural compounds (such as resveratrol [50], berberine [51], and curcumin [52]) have been identified as possible modifiers of LOX-1 expression. A more comprehensive approach and eventually a longitudinal study will be needed for further elucidation of the reported results.

## 5. Conclusions

In conclusion, we demonstrated that the serum levels of both cLDL and sLOX-1 were significantly increased in patients with MetS, but this increment was even more remarkable in subjects with MetS accompanied by CAD. In addition, a significant positive correlation between cLDL and sLOX-1 was established only in the MetS + CAD group. This suggested that the interaction between cLDL and LOX-1 might be involved in the pathogenesis of CAD and associated with the higher CAD propensity among the MetS population. Therefore, the measurement of serum cLDL and sLOX-1 might help in the diagnosis and the risk assessment of MetS as well as of CAD in MetS patients.

## Figures and Tables

**Table 1:** Baseline clinical characteristics of control and metabolic syndrome (MetS) groups with and without coronary artery disease (CAD).

Variables	Control	MetS	MetS + CAD	*p*-Value
	(*n* = 30)	(*n* = 30)	(*n* = 30)	
Sex, Male/Female (no.)	12/18	9/21	11/19	0.712 ^#^
Age (years)	45.6 ± 9.1	47.8 ± 6.4	51.1 ± 9.3 *	0.025
Body mass index (kg/m^2^)	22.8	29.2	29.6	<0.001
	(21.6–24.8)	(28.3–30.2) ***	(28.7–31.4) ***	
Waist circumference (cm)	83.9 ± 10.0	103.7 ± 10.5 ***	100.7 ± 15.1 ***	<0.001
ACEI or ARB (no.)	-	12	9	0.589 ^#^
Systolic BP (mmHg)	115.4 ± 6.6	127.7 ± 8.3 ***	128.6 ± 6.8 ***	<0.001
Diastolic BP (mmHg)	76.2 ± 4.1	82.1 ± 6.8 ***	81.7 ± 6.4 ***	<0.001
Fasting glucose (mmol/L)	4.71 ± 0.46	5.13 ± 0.96	5.56 ± 1.13 *	0.002
Total cholesterol (mmol/L)	4.3	5.35	4.91	<0.001
	(3.58–4.78)	(4.70–6.00) ***	(4.30–5.40) **	
Triglycerides (mmol/L)	1.03	1.35	1.27	<0.001
	(0.80–1.41)	(0.90–1.84)	(0.88–1.97)	
HDL-cholesterol (mmol/L)	1.14 ± 0.46	1.19 ± 0.17	1.18 ± 0.22	0.229
LDL-cholesterol (mmol/L)	2.65	3.33	2.8	0.002
	(1.95–2.99)	(2.73–4.01) ***	(2.20–3.56)	
Urea (mmol/L)	4.93 ± 1.25	4.81 ± 0.95	4.98 ±1.17	0.896
Creatinine (μmol/L)	78.5 ± 11.9	80.8 ± 10.4	82.2 ± 12.5	0.537
hs-CRP (μg/mL)	0.61	1.74	4.14	<0.001
	(0.20–1.04)	(1.03–3.67) ***	(1.38–6.02) ***	

Data are presented as mean ± SD or median (25th—75th percentile). Differences among the three groups were determined using ANOVA or Kruskal–Wallis tests followed by independent-samples t- and Mann–Whitney U-tests with Bonferroni corrections for multiple comparisons. Significance level versus control at * *p* < 0.05, ** *p* < 0.01 and *** *p* < 0.001, respectively; # *p*-value of Chi-square test. ACEI: angiotensin-converting enzyme inhibitor; ARB: angiotensin II receptor blocker; BP: blood pressure; HDL: high-density lipoprotein; LDL: low-density lipoprotein; hs-CRP: high-sensitivity C-reactive protein.

**Table 2:** Correlations between the serum levels of sLOX-1 and the baseline classical cardiovascular risk factors in patients with MetS (*n* = 60).

Variables	r_s_	*p*-Value
Body mass index (kg/m^2^)	0.238	0.068
Waist circumference (cm)	−0.039	0.765
Systolic BP (mmHg)	0.251	0.053
Diastolic BP (mmHg)	−0.083	0.529
Fasting glucose (mmol/L)	0.414	0.001 *
Total cholesterol (mmol/L)	0.074	0.576
Triglycerides (mmol/L)	0.031	0.812
HDL-cholesterol (mmol/L)	−0.273	0.035 *
LDL-cholesterol (mmol/L)	0.074	0.574
hs-CRP (μg/mL)	0.247	0.057

r**_s_**: Spearman’s correlation coefficient.; * indicates statistical significance.

**Table 3:** Sensitivity, specificity, cutoff values and area under the curve (AUC) of cLDL, LOX-1, and hs-CRP in the diagnosis of MetS among control and MetS subjects without CAD.

Variables	Sensitivity (%)	Specificity (%)	Cutoff	AUC (95% CI)	*p*-Value
cLDL (mg/L)	90.0	73.3	312	0.761	0.001
				(0.626–0.896)	
sLOX-1(pg/mL)	73.3	63.3	244	0.692	0.011
				(0.555–0.829)	
hs-CRP (μg/mL)	76.7	73.3	1	0.762	<0.001
				(0.637–0.886)	

**Table 4:** Sensitivity, specificity, cutoff values, and AUC of cLDL, LOX-1, and hs-CRP in the diagnosis of CAD among all MetS patients.

Variables	Sensitivity (%)	Specificity (%)	Cutoff	AUC (95% CI)	*p*-Value
cLDL (mg/L)	86.7	63.3	475	0.811	<0.001
				(0.698–0.924)	
sLOX-1(pg/mL)	70.0	63.3	495	0.739	0.001
				(0.611–0.867)	
hs-CRP (μg/mL)	56.7	77.7	3	0.658	0.035
				(0.519–0.798)	
